# Directed evolution of bacteriophages: thwarted by prolific prophage

**DOI:** 10.1128/aem.00884-24

**Published:** 2024-10-30

**Authors:** Tracey Lee Peters, Jacob Schow, Emma Spencer, James T. Van Leuven, Holly Wichman, Craig Miller

**Affiliations:** 1 Institute for Modeling Collaboration and Innovation, University of Idaho, Moscow, Idaho, USA; 2 Department of Biological Sciences, University of Idaho, Moscow, Idaho, USA; 3 Department of Animal, Veterinary and Food Sciences, University of Idaho, Moscow, Idaho, USA; Danmarks Tekniske Universitet The Novo Nordisk Foundation Center for Biosustainability, Kgs. Lyngby, Denmark

**Keywords:** bacteriophage, prophage, directed evolution, Appelmans, host range, *Pseudomonas aeruginosa*

## Abstract

Various directed evolution methods exist that seek to procure bacteriophages with expanded host ranges, typically targeting phage-resistant or non-permissive bacterial hosts. The general premise of these methods involves propagating phage(s) on multiple bacterial hosts, pooling the lysate, and repeating this process until phage(s) can form plaques on the target host(s). In theory, this produces a lysate containing input phages and their evolved phage progeny. However, in practice, this lysate can also include prophages originating from bacterial hosts. Here, we describe our experience implementing one directed evolution method, the Appelmans protocol, to study phage evolution in the *Pseudomonas aeruginosa* phage–host system, where we observed rapid host-range expansion of the phage cocktail. Further experimentation and sequencing revealed that the observed host-range expansion was due to a *Casadabanvirus* prophage originating from a lysogenic host that was only included in the first three rounds of the experiment. This prophage could infect five of eight bacterial hosts initially used, allowing it to persist and proliferate until the termination of the experiment. This prophage was represented in half of the sequenced phage samples isolated from the Appelmans experiment, but despite being subjected to directed evolution conditions, it does not appear to have evolved. This work highlights the impact of prophages in directed evolution experiments and the importance of genetically verifying output phages, particularly for those attempting to procure phages intended for phage therapy applications. This study also notes the usefulness of intraspecies antagonism assays between bacterial host strains to establish a baseline for inhibitory activity and determine the presence of prophage.

**IMPORTANCE:**

Directed evolution is a common strategy for evolving phages to expand the host range, often targeting pathogenic strains of bacteria. In this study, we investigated phage host-range expansion using directed evolution in the *Pseudomonas aeruginosa* system. We show that prophages are active players in directed evolution and can contribute to observation of host-range expansion. Since prophages are prevalent in bacterial hosts, particularly pathogenic strains of bacteria, and all directed evolution approaches involve iteratively propagating phage on one or more bacterial hosts, the presence of prophage in phage preparations is a factor that needs to be considered in experimental design and interpretation of results. These results highlight the importance of screening for prophages either genetically or through intraspecies antagonism assays during selection of bacterial strains and will contribute to improving the experimental design of future directed evolution studies.

## INTRODUCTION

Directed evolution of phages is a term used to describe the experimental methods that promote genetic and phenotypic evolution of phages toward some desired trait. Directed evolution methods vary by the host presentation strategy and typically fall into three categories: sequential, parallel, or mixed (1). In all cases, the general strategy of directed evolution methods includes iteratively propagating phages on one or more hosts, then harvesting the output phage lysate after growth, and using it as the input phage(s) for the following round. Also referred to as phage training, one goal of directed evolution is to procure phages with altered or expanded host range that can infect phage-resistant or otherwise non-permissive hosts (2). When this outcome is achieved, it is often the result of phage accumulating point mutations and/or a recombination event in tail fiber and receptor-binding genes (2–6). However, some studies have shown that epigenetic modifications, particularly DNA methylation, can contribute to changes in the phage host-range as phages adapt to evade bacterial defense systems like restriction–modification. Phages can encode orphan DNA methyltransferases or alter their methylation patterns to overcome anti-phage defense systems, thereby enhancing their infectivity toward new hosts (7–10).

The Appelmans protocol is one directed evolution method that is used to expand the host range of bacteriophages for phage therapy applications (2, 11, 12). This method employs a parallel presentation of bacterial hosts to a phage cocktail of two or more phages, where the phage mixture is serially diluted and allowed to propagate on individual hosts, followed by harvesting the pooled lysate. The pooled phage lysate is then again diluted and allowed to propagate on individual hosts for a variable number of subsequent iterations (2, 11). The end goal is to obtain a phage lysate or an individual phage isolate that has evolved to infect a desired number of target bacterial hosts. Subsequent infections increase the likelihood of coinfection and recombination, thus increasing the potential for diversity in the resulting pooled phage lysate, without the addition of new genetic information (4).

In theory, as the input phage cocktail is propagated on the hosts, the pooled lysate includes both input and evolved phage progeny. In practice, the basic methodology for harvesting the pooled phage lysate in subsequent rounds results in the inclusion of input phages, their potentially evolved progeny, and inevitably, various cellular components and bacterial byproducts, such as resident prophage induced from the bacterial host(s).

Many pathogenic bacterial strains of clinical importance carry prophage gene clusters within their genomes (13–15), which can include cryptic prophages, tailocins, and filamentous phages (16–19). Exposure of a prophage-carrying host (otherwise known as a lysogen) to mitomycin C or ultraviolet light can often lead to prophage induction, although some prophages may be produced at low levels (previous studies reported induction rates of 0.09% to 3.1% of the bacterial population) due to spontaneous DNA damage and heterogenous expression of genes involved in the SOS response (20, 21). Prophages may be maintained by the host because they can contribute some advantages pertaining to survival and fitness such as promoting biofilm formation, bacterial virulence, immunity to subsequent phage infections, and horizontal gene transfer (22–25). Thus, the presence of prophage in bacterial hosts can be considered a source of additional “surprise” genetic input in directed evolution conditions.

Although the prevalence of prophages is well established and directed evolution methods are common practice, the phenomenon of prophage induction during experimental evolution of phages and the potential influence on experimental outcomes or data interpretation has been underreported until recently. For example, Vu et al. found that prophage induction and evolution occurred during the Appelmans protocol in the *Acinetobacter baumanni* phage–host system and contributed to the expanded host range of the phage cocktail (26). Reporting and acknowledging the presence and influence of prophages in directed evolution experiments will contribute to improved experimental design in future studies.

Here, we describe our experience employing the Appelmans protocol to study phage evolution on *Pseudomonas aeruginosa*, in which prophage induction early in the experiment was responsible for the observed rapid host-range expansion. The prophage originated from a clinical isolate strain, which was only included in the first three rounds of the Appelmans protocol (out of nine total rounds). This strain was then removed from the experiment due to slow growth rates. The wild-type prophage had the ability to infect five target bacterial hosts, allowing it to proliferate and persist through the end of the experiment, despite its host of origin being removed. This prophage remained at high abundance, outcompeted the input phages, and was represented in half of the phage samples isolated from the experiment, as confirmed by whole-genome sequencing. Further genetic and host range analyses suggest that although this prophage was subjected to directed evolution conditions, it did not evolve to expand the host range over the course of the experiment. The low frequency of mutations observed over time suggests that this phage is genetically stable (27, 28). This phage was found to be closely related to a known lysogenic, generalized transducing phage, JBD24 of the genus *Casadabanvirus* (29). This work highlights the importance of genetically verifying input and output phages in directed evolution studies and serves as a cautionary tale for future design of directed evolution experiments, particularly for those attempting to procure phages for phage therapy applications (30). We also outline the utility of intraspecies antagonism assays for identifying underlying cross-strain activity due to tailocins or resident prophages.

## RESULTS

### Using directed evolution to expand the phage host range

**Fig 1:**
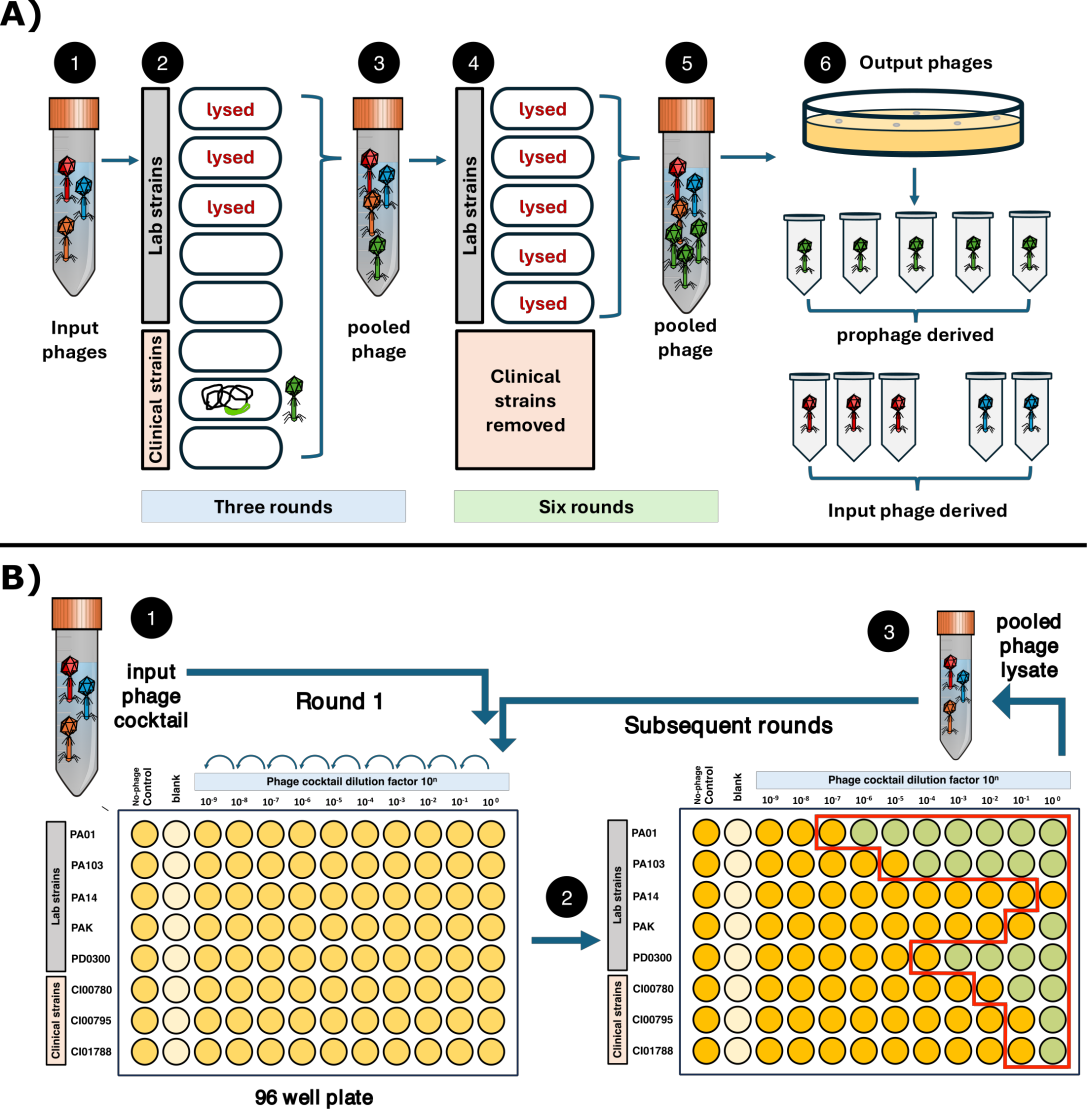
(**A**) Summary of the experimental design and results: The input phage cocktail consisted of three phages (1) that could collectively infect and lyse three of the target bacterial hosts used in the Appelmans protocol (2). A prophage was induced from a clinical isolate strain within the first three rounds (3) that could infect five of the target strains (4). The three clinical isolates were removed from Appelmans due to slow growth rates after the third round. The prophage persisted through the ninth (final) round of Appelmans (5) and comprised half of the sequenced phage samples isolated from the experiment (6). (**B**) Experimental layout of the Appelmans protocol in a 96-well plate. (1) The input phage cocktail is diluted and applied to each bacterial host; (2) overnight incubation results in lysed, cleared wells (green) and unlysed, uncleared wells (yellow); (3) all lysed and the first unlysed well are pooled (red outline) and used as the input phage cocktail for subsequent rounds.

The purpose of this work was to study the genetic determinants of phage evolution in the context of phage host-range expansion. The goal was to expand the host range of three input phages onto non-permissive hosts and then identify genetic mutations that may have contributed to the phenotypic change. Thus, we endeavored to implement the Appelmans protocol in *Pseudomonas aeruginosa,* a human pathogen given high research priority (see Fig. 1 for experimental design) (2). Three temperate phages were used as the input phage cocktail: D3, M6, and JM2 (31, 32). Phage D3 is of the genus *Detrevirus*, while phages M6 and JM2 are of the genus *Yuavirus*. Early studies with D3 suggest that this phage binds to the cell wall, particularly the O-antigen of LPS, and can cause serotype conversion of the host following lysogeny, as a defense against closely related, superinfecting phages (33–35). M6 and JM2 share a whole-genome nucleotide identity of 91%, but an identity of <25% with phage D3. Thus, recombination may be expected between M6 and JM2, but is unlikely with D3. Early studies with M6 demonstrated that this type of phage uses pili as an initial receptor and is drawn to the cell surface upon pilus retraction, where contact with the cell surface initiates DNA injection (36). Five laboratory strains (three permissive and two non-permissive) and three clinical isolates (non-permissive) were originally used for the first three rounds of the protocol. Preliminary host-range analysis of the input phages established that they could each infect two hosts, PAO1 and either PA103 or PDO300, collectively infecting three of eight host strains initially selected. Phages were tenfold serially diluted and added to the respective wells for each bacterial host. After overnight incubation, all lysed, cleared wells and the first unlysed, uncleared well for each host were pooled, and the lysate was prepared as the input phage cocktail for the subsequent round.

### Rapid host-range expansion and lysis of non-permissive hosts

**Fig 2:**
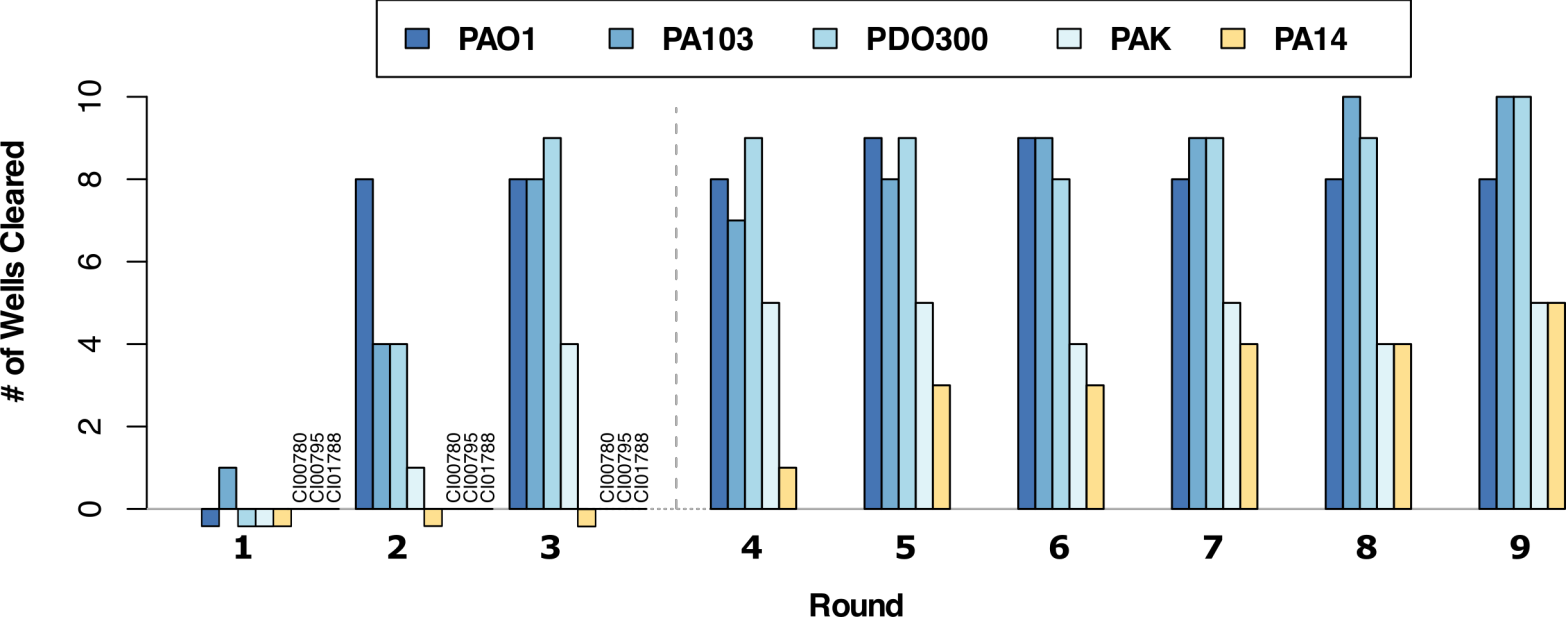
Directed evolution of phages resulted in rapid host-range expansion of the phage cocktail. Eight strains were individually challenged with a cocktail of three input phages for the Appelmans protocol for nine rounds. Clinical isolates strains CI00780, CI00795, and CI01788 were removed from the experiment after round 3 due to slow growth rates, designated by the dashed line. Lysis of non-permissive hosts PAK and PA14 was observed by round 4.

**Fig 3:**
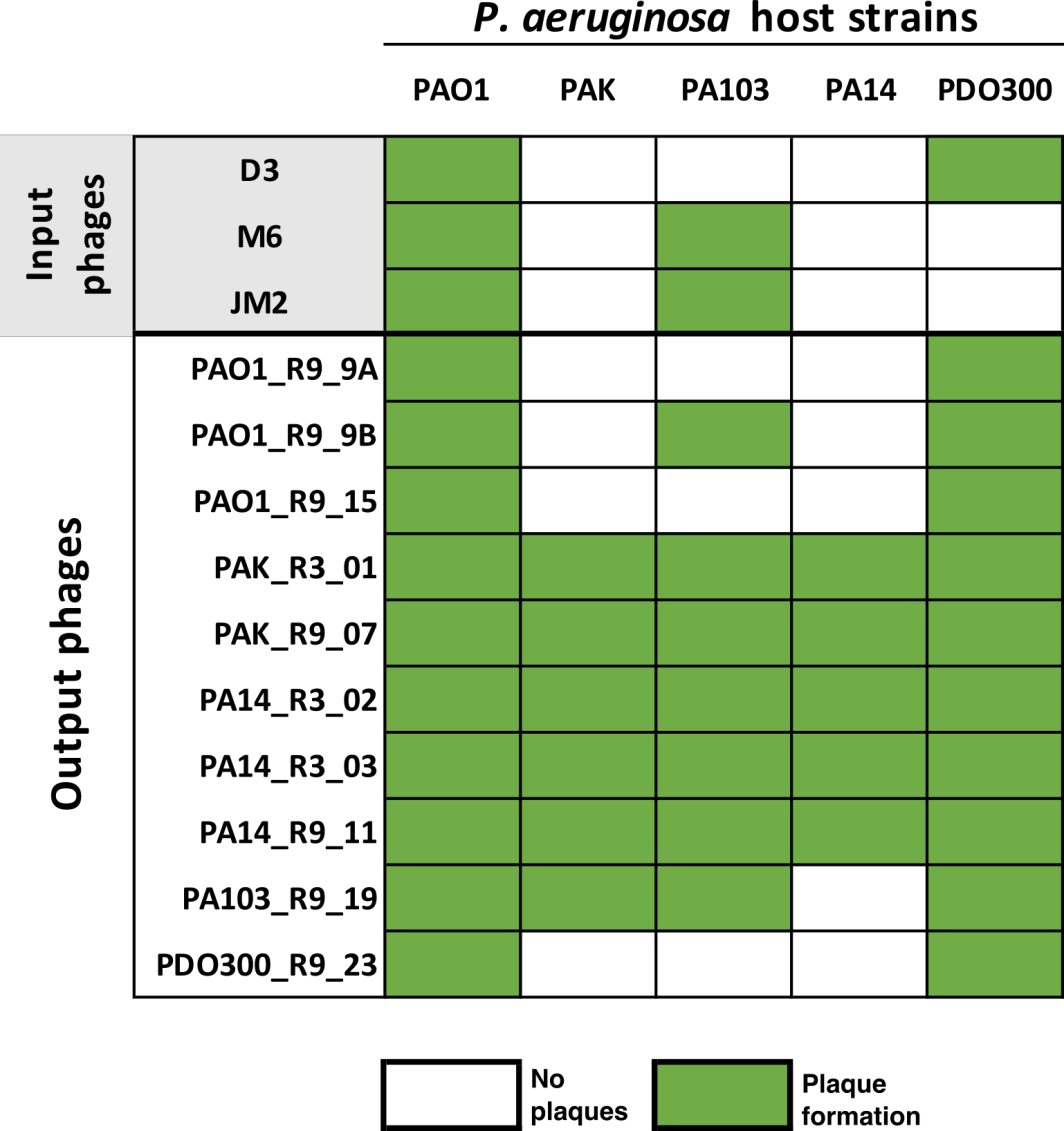
Host range of phages. Matrix representing the host range of input phages and output phages isolated from Appelmans. The output phage naming scheme designates isolation host, isolation round, and phage number.

The Appelmans protocol was implemented for a total of nine rounds. Surprisingly, host-range expansion of the phage cocktail was observed within the first four rounds of passaging, with clearing of non-permissive hosts PAK (in the second round) and PA14 (in the fourth round) (Fig. 2). The three clinical isolates were removed from the experiment after the third round due to slow growth rates compared to the laboratory strains. To assess changes in the host range of the evolving phage cocktail, the pooled phage lysate was plated after rounds 3 and 9. The pooled phage lysate was plated onto lawns of target strains, and resulting phage plaques were re-isolated by double plaque purification on individual hosts. Host-range analysis of output phages showed that five phage isolates with an expanded host range could form plaques on all five target hosts, PAO1, PA103, PDO300, and non-permissive hosts PAK and PA14 (Fig. 3; Fig. S1, panel A). Three of these phages were isolated after round 3, and two were isolated after round 9 on either strain PAK or PA14. No plaques were observed on the clinical isolate strains. Ten output phages of interest were prepared for DNA extraction and submitted for sequencing.

### Output phages with expanded host range were not derived from input phages

Output phages were sequenced using an Illumina Miseq platform, and their genomes were *de novo* assembled using UniCycler. Output phage genomes were then compared to the three input phage genomes, D3, M6, and JM2. Phages D3, M6, and JM2 have genomes of approximately 56 kbp, 61 kbp, and 60 kbp, respectively (Fig. S2). Surprisingly, assemblies for the five output phages with an expanded host range (Fig. 3: PAK_R3_01, PAK_R9_07, PA14_R3_02, PA14_R3_03, and PA14_R9_11) produced contigs between 37 kbp and 39 kbp, none of which were derived from input phages. Thus, we hypothesized that these output phage isolates may be derived from prophages originating from one of the bacterial hosts used in Appelmans.

### Intraspecies antagonism assays revealed the presence of prophage in two host strains

**Fig 4:**
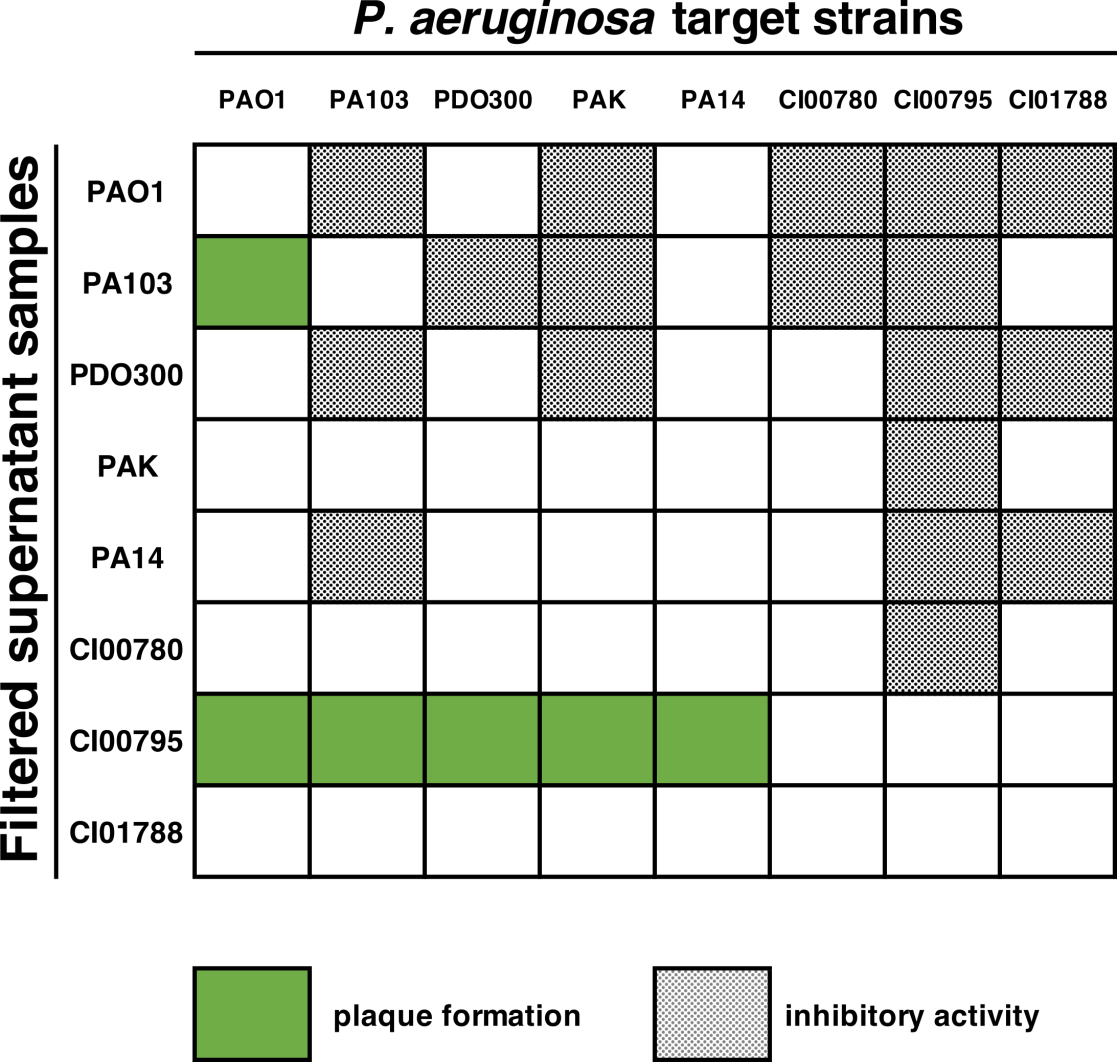
Intraspecies antagonism assay showed the presence of active prophage in strains PA103 and CI00795. Inhibitory activity was observed from six out of eight hosts.

To test the hypothesis that output phages may be derived from prophage, we conducted intraspecies antagonism assays between hosts used in Appelmans. All eight *P. aeruginosa* strains originally used in the experiment were cultured and subjected to induction by ultraviolet light and mitomycin C. Intraspecies antagonism was measured by removing cells from these cultures and spotting the supernatants on lawns of all eight *P. aeruginosa* strains. Plaque formation was observed from the supernatant of strains PA103 and clinical isolate CI00795 (Fig. 4). We also observed cleared zones of inhibition (Fig. S1, panel B) from six out of eight strains, which is characteristic of tailocin activity (also known as pyocins in *Pseudomonas*) (37, 38). Exposure of cultures to inducing agents did not produce higher levels of plaque formation or zones of inhibition compared to unexposed cultures (Fig. S1, panel C), suggesting that these prophages are spontaneously produced from the hosts at low levels. Supernatants from PA103 could only form plaques on strain PAO1, and plaque formation was eradicated when the sample was exposed to chloroform (Fig. S1, panel D). The prophage from PA103 may also be induced by exposure to heat, as lawns made from this host were riddled with plaques. Supernatants from strain CI00795 were able to form plaques on all five laboratory strains, including the two non-permissive hosts PAK and PA14, and were not susceptible to chloroform (Fig. S1, panel E). Considering the narrow host range of the PA103 prophage and that chloroform was regularly used to harvest and isolate phages throughout the Appelmans experiment, we determined it unlikely that the PA103 prophage contributed to host-range expansion. Thus, we selected the clinical isolate strain CI00795 for further analysis and sequencing.

### Five output phages are derived from a *Casadabanvirus*-like prophage in strain CI00795

**Fig 5:**
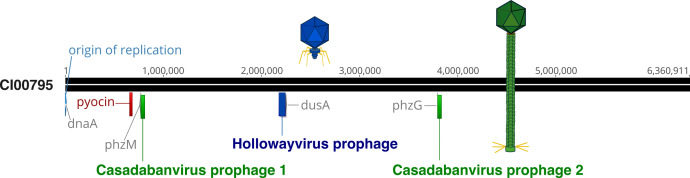
Genome map of *Pseudomonas aeruginosa* strain CI00795. The genome is oriented to begin with the origin of replication. The *Casadabanvirus* prophage is integrated at two locations: once near the pyocyanin biosynthetic protein (phzM) and again near a phenazine biosynthesis FMN-dependent oxidase (phzG). The Hollowayvirus prophage is integrated near tRNA dihydrouridine synthase (dusA). A pyocin gene cluster is also present, located between anthranilate synthase component I (trpE) and anthranilate synthase component II (trpG).

To determine the origin of the five phage isolates with expanded host range, whole-genome sequencing and hybrid assembly of the clinical host strain CI00795 genome were performed. Hybrid assembly using both short- and long-read data resulted in a single, circular contig of 6,360,911 bp (NCBI accession: CP158022, coverage: 234.7×). Genomic analysis of the CI00795 genome revealed that this host carries two prophages, one that is approximately 70 kbp and a second that is 37 kbp (Fig. 5). The 70-kbp prophage is a *Hollowayvirus*-like prophage and is integrated between genes annotated as an exo-alpha-sialidase and a tRNA dihydrouridine synthase (dusA) (nucleotide position 2,176,677 to > 2,240,360). The 37-kbp prophage is represented twice in the host genome, integrated in two locations: once between the genes phzM and hxlR (nucleotide positions 771,168 to > 808,371, henceforth referred to as “phi_1”) and again between phzG and an ORF annotated as a DNA excision repair complex subunit (nucleotide position 3,795,192 to > 3,832,395, henceforth referred to as “phi_2”). Sequence alignments confirmed that the five output phage samples were derived from the 37-kbp prophage.

**Fig 6:**
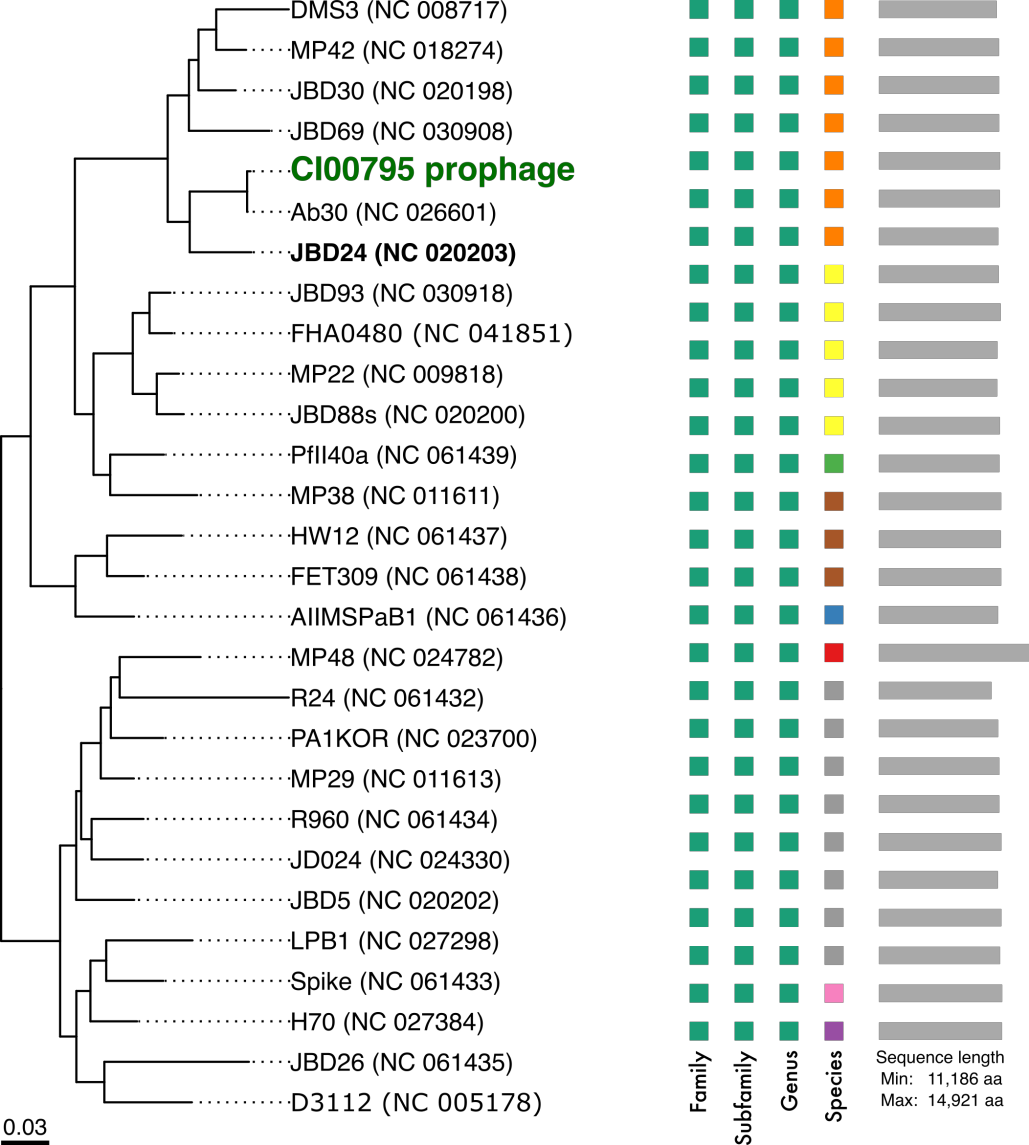
Phylogeny of the CI00795 37-kb prophage within the *Casadabanvirus* genus, consisting of 27 members as listed by ICTV. The tree is inferred from the amino acid sequences obtained from NCBI reference genomes using the VICTOR pipeline with the GBDP d6 formula.

Further genomic analysis of the CI00795 prophages phi_1 and phi_2 indicated that this phage belongs to the genus *Casadabanvirus* (Fig. 6), with 88.9%–99.9% whole-genome average nucleotide identity using Blastn against 27 *Casadabanvirus* NCBI reference genomes based on the current genus listed by the International Committee for Taxonomy of Viruses. Phages in this genus, such as JDB24, have linear dsDNA genomes and are reported to be temperate, transposable, transducing, phages that, upon lysogenizing a host, can enhance the plaque formation of other infecting phages that are sensitive to CRISPR (29). They also integrate into multiple locations by transposition and can package up to 2 kbp of heterogenous host DNA on the ends of their genome (39). Extraction and comparative genomics of phi_1 and phi_2 from the genome of CI00795 revealed that they are approximately 37,204 kbp and vary by six nucleotides (Fig. S3; Table S1). Variant analysis of the five output phages derived from the *Casadabanvirus* prophage revealed that four of the samples shared 100% identity with each other (excluding heterogenous host DNA regions) (Fig. S3), with the fifth sample sharing 99.99% identity. Only two unique mutations were identified in the five output phages that were not present in the two integrated prophages phi_1 and phi_2. All five output phages housed an intergenic insertion of “G” in a 5-nucleotide homopolymeric tract upstream of the tape measure protein. The fifth sample, PA14_R9_11, housed a nonsynonymous mutation in a putative tail fiber or baseplate protein. Three of these phages were isolated after round 3 (PAK_R3_01, PA14_R3_02, and PA14_R3_03), and the other two were isolated after round 9 (PAK_R9_07 and PA14_R9_11). Considering that the frequency of mutations for this phage remained low over the course of nine rounds of directed evolution conditions, this indicates that this phage is genetically stable (27, 28).

### Host-range expansion of the *Casadabanvirus* prophage did not occur

Having established the origin of the five output phage samples, we sought to compare their host range with the initial host range of the *Casadabanvirus* prophage. To achieve this, we first plated the lysate from strain CI00795 using the double agar overlay method against each of the strains used in Appelmans to observe plaque formation. We then aimed to determine the host range of the *Casadabanvirus* prophage and the Hollowayvirus prophage. To do this, we designed PCR primers that targeted the large terminase subunit of either prophage. Individual plaques were then isolated from each host strain and subjected to PCR analysis using both sets of primers. This approach allowed us to distinguish the host range of each prophage in CI00795: the *Hollowayvirus* prophage can form plaques on PAK and PDO300, but no positive results were observed for plaques harvested from the other strains. The *Casadabanvirus* prophage was confirmed to form plaques on host strains PAO1, PA103, PA14, PAK, and PDO300 (Table S2), with no evidence for double positives. These results indicate that the wild-type *Casadabanvirus* prophage could initially infect all five hosts, and although it was subjected to directed evolution conditions, it did not undergo evolution that led to an altered host range against the host strains tested.

### Three output phages are derived from input phage D3, and two output phages are derived from JM2

Further analysis of the five remaining output phages isolated from the Appelmans experiment showed that three phages (PAO1_R9_9A, PAO1_R9_15, and PDO300_R9_23) were derived from input phage D3, and two (PAO1_R9_9B and PA103_R9_19) were derived from input phage JM2. The three output phages derived from D3 were 100% identical to each other, differing from the input phage D3 only by a 19-bp insertion in the N-terminal region of a CI-like transcriptional repressor gene (locus tag D3p074, D3 genome NC_002484) at residue 36/224 (Fig. S3A). SMART analysis predicted a helix-turn-helix, DNA-binding domain at residues 6–60. This insertion disrupts the reading frame and suggests the altered function of the final product. Insertions into the N-terminal of the CI repressor have previously been shown to inactivate the repressor and allow for transcription of lytic genes (40). Although the host range of D3-derived output phages did not differ from the input phage, it is possible that these output phages are more virulent due to mutations in the CI repressor. Future work will investigate the effects of this mutation.

The two JM2-derived output phages are identical to the input phage, aside from mutations in a gene annotated as a structural protein (locus tag PPM6_gp033 in M6 genome NC_007809). PA103_R9_19 and PAO1_R9_9B share one amino acid change at residue 178/331 (Ser->Arg). PAO1_R9_9B also houses a 154-bp recombination region within this gene, which is derived from input phage M6 (Fig. S3B). This recombination event results in nine amino acid substitutions within this region compared to the JM2 input phage. PHYRE2 predictions suggest this protein may be part of the baseplate complex (RCBS:8rk3) and was modeled based on the template for the tail fiber protein gp48 of *Pseudomonas* phage JBD30 (PDBe) (41) with 100% confidence 90%. Valentova *et al*. recently reported that gp47 is part of the baseplate of phage JBD30 and forms a heterodimer with gp48 to form a tail fiber that binds to Type IV pili. They suggest that this interaction may orient and transport the phage to the cell surface by pilus retraction, followed by attachment of the receptor-binding proteins (41). This is consistent with previous work on phage M6, which identified that this phage interacts with pili as the first step in adsorption to the host cell surface (36). The host range of PAO1_R9_9B was expanded to include only one additional host (PDO300) compared to input phages JM2 and M6 (infect PAO1 and PA103), whereas PA103_R9_19 evolved to infect both PDO300 and PAK. Future work will test the host ranges of these evolved phages on a larger panel of *P. aeruginosa* strains to understand the extent that these genetic changes have on the host range.

## DISCUSSION

*“In theory, there is no difference between theory and practice, while in practice there is.”*

*-* Benjamin Brewster (42)

The directed evolution of phages involves methods aimed at promoting genetic and phenotypic variations to procure phages with desired characteristics. In theory, input phages propagated on input hosts will result in evolved input phages with the desired trait, and these dynamics can be modeled and outcomes predicted (1). However, in practice, directed evolution studies can lead to variable and unexpected outcomes due to the complex dynamics of phage–host interactions and experimental conditions. Prophages, dormant viral genomes housed within bacterial hosts, can influence these outcomes as an unexpected input and contribute to misinterpretation of results when not taken into consideration. This phenomenon poses challenges for some applications, while offering solutions for others. For instance, in phage–host systems where lytic phages are scarce, the presence and induction of prophages can be viewed as a resource of potential therapeutic options. However, unwanted prophage content complicates the isolation of evolved phages and may impact the regulatory approval of phages for therapeutic applications.

Here, we showed that induction of a prophage occurred during directed evolution in the *P. aeruginosa* phage–host system. The presence of this prophage was responsible for the observed host-range expansion of the pooled phage lysate onto previously non-permissive hosts. This prophage had a broader host range than the input phages, which allowed it to persist throughout the experiment, outcompete input phages, and become the most commonly isolated phage, even though the host it originated from was removed from the experiment after round 3. Although the prophage was subjected to directed evolution conditions, it does not appear to have undergone host-range expansion. Although the induction and proliferation of prophage under directed evolution conditions were unexpected, it is not necessarily a surprising result, given the nature and prevalence of prophages.

The number of prokaryotic genomes available has increased exponentially over the last two decades (43). This coupled with the ever-growing number of prophage prediction programs has allowed for the identification of prophage gene clusters in many bacterial species. Prophages are highly prevalent in human pathogens in particular, with some strains harboring multiple prophages (15, 44–47). Furthermore, recent studies focused on the directed evolution of phages reported the induction of prophages. In the *Acinetobacter baumanii* phage–host system, prophages were observed to evolve via recombination, which resulted in a prophage with an expanded host range (26). In the *Klebsiella* phage–host system, prophage induction was only observed when cultures were exposed to all three input phages, but not when cultures were exposed to individual phages (48). These findings, along with those of the current study, provide three examples of induction of prophage from pathogenic bacterial hosts during directed evolution. There is also a dissertation from Peter Dougherty that states in the abstract that phage isolates with an expanded host range were descended from prophages (49).

Prophage induction and proliferation under directed evolution conditions is a fascinating, albeit mildly inconvenient, phenomenon; it presents a concerning problem for some, but a potential solution for others. For example, in some phage–host systems, strictly lytic phages are scarce, and only temperate phages are available for biocontrol or therapeutic applications (50, 51). If directed evolution methods can create conditions that allow for the induction and recombination of prophages, this may be a useful approach to procure evolved temperate phages with the desired host range. The Appelmans protocol was previously shown to encourage host-range expansion through recombination in the *Pseudomonas* phage–host system (2). Lossouarn et al. (4) also demonstrated that the Appelmans protocol promoted point mutations and recombination events in tail-related genes in the *Enterococcus faecium* phage–host system, resulting in host-range expansion from 35% to 70% of their targeted strains in 15 rounds (4). Although we did not observe any recombination events or host-range expansion in the prophage-derived phage isolates, we did observe one recombination event and a point mutation in the JM2-derived phages, both located in a putative tail fiber gene that is likely involved in binding to type IV pili (41). This recombination event occurred between M6 and JM2, the two temperate input phages that had >90% sequence identity, demonstrating that directed evolution conditions such as Appelmans can encourage recombination events between closely related phages. Although the use of temperate phages for phage therapy has been controversial, this approach is no longer out of reach in light of the advancements in genetic engineering (52–54). Directed evolution methods, such as Appelmans, may present an opportunity to further study the evolution, induction pathways, and phage–host dynamics of prophages and temperate phages that could expand the pool of phages available for therapeutic applications.

In the current work, the prophage induced from a clinical isolate strain during Appelmans would be considered an interesting problem as *Casadabanvirus* phages are known transposable, transducing, lysogenic phages that would not be suitable for phage therapy applications. Although the presence of *Casadabanvirus* phages in host genomes has been shown to improve the virulence of superinfecting phages otherwise sensitive to CRISPR, such as is the case with phage JBD24 (55), it has also been shown that polylysogeny with this phage can lead to increased phage resistance of the lysogenized host (29). Prophages are known to carry anti-phage defense mechanisms that can benefit their host (25); however, some phages can also interfere with these systems. For example, *Casadabanvirus* phage DMS3 can block anti-phage defense mechanisms in the host, as well as block pilus assembly, inhibiting phage that use the pilus as a receptor (56). If interfering with pilus assembly is a common strategy for phages in the *Casadabanvirus* genus to excluded other phages from infecting*,* it is possible that the CI00795 prophages were able to exclude pilus-dependent phages M6 and JM2 from infecting (36).

Considering the prevalence of prophages in clinical isolates and that directed evolution is commonly applied to target strains of clinical significance, the issue of prophage content is one that warrants greater attention. This raises the question: how does one solve the problem of unwanted prophage content? It is clear that sequencing input phages and evolved output phages is the most reliable method of ensuring that evolved phage isolates are derived from input phages, rather than being derived from prophages. Otherwise, the genetic origin of “evolved” phages isolated from directed evolution will remain unknown (2). The sequencing of bacterial strains is also important, particularly for those strains used for phage propagation and phage isolation. Having complete genome sequences for the host is extremely useful in understanding resident phage-related elements that could influence Appelmans results, such as tailocins and prophages. However, sequencing all bacterial target strains is not practical or feasible for many due to resource constraints or time.

Here, we were able to utilize intraspecies antagonism assays of bacterial hosts to screen for potential prophage and tailocin content. This is a useful approach to establish a baseline of cross-strain inhibitory activity, including the host range of resident prophage. Conducting this assay prior to implementing Appelmans may also be helpful in selecting a suitable panel of propagation and target host strains. These data are also useful for interpretation of phage host-range testing of putatively evolved phages. Another approach could be to run a phage-free control of Appelmans to see if lysis occurs due to induction of tailocins or resident prophage. Here, we were also able to use PCR to efficiently screen plaques to identify and determine the host range of resident prophages. Alternatively, primers that are specific to input phages could be designed and PCR used to quickly correlate the observed plaque formation of the pooled phage lysate with phage of origin to avoid pursuing unwanted prophage-derived isolates and preserve sequencing resources.

In conclusion, the presence and proliferation of prophages during directed evolution experiments present both obstacles and opportunities in the quest to procure phages with desired characteristics for therapeutic applications. Such studies deepen our understanding of fundamental phage–host interactions. Understanding and managing the presence and influence of prophages in directed evolution studies is important for experimental design, allocation of resources, proper interpretation of results, and increasing the likelihood of favorable outcomes. Strategies such as sequencing input and output phages, sequencing hosts, performing intraspecies antagonism assays, and utilizing PCR for phage identification can aid in addressing these challenges.

## MATERIALS AND METHODS

### Bacterial and bacteriophage strains

All bacterial and bacteriophage strains are listed in Table S3. All bacteria and phage were maintained at −80°C in 750 μL of LB media with 30% (wt/vol) glycerol. Bacterial strains were prepared for use by streaking for isolated colonies on 1.5% (wt/vol) LB agar plates and incubating at 37°C. Single colonies were used to prepare overnight cultures by inoculating 3 mL of LB media and incubating with shaking at 37°C. All input phages were propagated on host strain PAO1 Seattle. The host range of input phages was determined by spot plating 10 μL of serially diluted phage stock onto lawns of bacterial hosts prepared on 3 mL LB top agar (0.7% wt/vol), followed by incubation of plates at 37°C overnight and observation of plaque formation.

### Appelmans method

The Appelmans protocol is a method for passaging a phage cocktail on multiple hosts simultaneously, typically in a 96-well plate (2). The experiment was set up as previously described by Burrowes et al. (2) Briefly, bacterial hosts were arranged in a 96-well plate by row and evenly distributed across columns by adding 1 μL of culture to 100 μL of double strength LB media (Fig. 1A). The input phage cocktail contained a 1:1:1 ratio of phages D3, M6, and JM2, with a total input titer of 1 × 10^7^ PFU/mL. Tenfold serial dilutions of the phage cocktail were made in LB media, and 100 μL of each dilution was added to wells across columns, for a final volume of ~200 μL. The plate was then incubated overnight at 37°C and 200 rpm. After overnight incubation, lysis of wells was observed and documented. All lysed wells and the first unlysed or turbid well were collected in a 5-mL Falcon tube and chloroformed with 1:10 (vol:vol) ChCl3. The pooled phage lysate was centrifuged for 10 minutes at 10,000 × *g*, and the supernatant was transferred and used as the input phage cocktail for the following round, for a total of nine rounds.

### Isolation of phages from Appelmans

The pooled phage lysate from Appelmans was serially diluted and plated on lawns of *P. aeruginosa* after round 3 and round 9. Resulting plaques from the Appelmans protocol were double plaque purified by plating dilutions of the cocktail on each host individually using the top agar overlay method [overnight host culture and phage lysate dilution suspended in 3 mL of LB top agar (0.7% wt/vol)]. A plug of agar in the center of an individual plaque was taken and resuspended in a microcentrifuge tube containing 750 μL LB media and 50 μL ChCl3. This tube was then vortexed and spun down at 10,000 × *g* for 10 minutes before the supernatant was collected. This process was repeated with the single isolated plaque supernatant to form a double isolated plaque supernatant. This 2× isolated plaque in LB media was then used as the working stock for subsequent host-range assays.

### Host-range analysis of phages isolated from Appelmans

The host range of output phages was determined by observation of plaque formation on indicator lawns of bacteria; plaque formation indicates productive phage infection. To visualize plaques, 100 μL of the bacterial host culture was plated using the LB top agar overlay method to create a bacterial lawn. The top agar was allowed to set for 5–10 minutes before 2 μL of phage dilutions was spotted across the lawn. Using a flame-sterilized tungsten tool, each spot was streaked horizontally across the plate, flaming the tool between each use. After streaking, plates were incubated for 4–5 hours at 37°C and then left at room temperature for ~8 hours before analysis. Streaks that resulted in individual plaque formation were considered positive.

### Bacterial and phage genomic DNA extraction and sequencing

Bacterial gDNA for strain CI00795 was extracted using the QIAgen DNeasy UltraClean Microbial kit as per the manufacturer’s instructions. Phage stocks were amplified to a titer of ~1×10^10^ PFU/mL by plating 1 × 10^5^ PFU/mL with 100 μL of the PAO1 Seattle overnight culture by LB top agar overlay. After overnight incubation at 37°C, the top layer of the cleared plate was collected in a 5-mL microcentrifuge tube. After addition of 1:10 (vol:vol) ChCl3, 1.5 mL of LB media, the tube was spun down at 10,000 × *g* for 10 minutes, and 1 mL of the supernatant was transferred to a separate 5-mL microcentrifuge tube. Phage gDNA was extracted using the Norgen Phage DNA Extraction Kit (including the optional protease and second elution steps). Bacterial and phage gDNA stocks were stored at −20°C. Bacterial and input parent phage samples were submitted for sequencing with Omega (Norcross, GA). Evolved phage samples were submitted for sequencing with SeqCoast (Portsmouth, NH). Samples were prepared for whole-genome sequencing using an Illumina DNA Prep tagmentation kit and unique dual indexes. Sequencing done with Omega was conducted on an Illumina HiSeq platform. Sequencing done with SeqCoast was performed on the Illumina NextSeq2000 platform. At least 2 million 150 bp paired-end reads were obtained for every phage isolate, and 5 million 150 bp paired-ends reads were obtained for the bacterial sample CI00795. Strain CI00795 was also submitted to Plasmidsaurus for bacterial gDNA extraction and sequencing using Oxford Nanopore Technology.

### Assembly, genomic, and variant analysis of bacterial hosts and phage isolates

Illumina reads were trimmed using fastp (version 0.22.0) (57). FastQC was used to check for the read quality of Illumina reads (version v0.11.3) (58). Reads were assembled using Unicycler (59) (version v0.4.3); a hybrid assembly was constructed for bacterial isolate CI00795 using the trimmed Illumina data set and the long read data set from Plasmidsaurus. The bacterial genome was annotated using Bakta (60). Phage genomes were annotated using pharokka (61). Assembly statistics were generated using minimap2 (62) and samtools (63). Variant analysis was conducted using breseq (64) and pairwise and multiple sequence alignments using Blastn and Geneious Prime, respectively (version 11.0.15 + 10) (65). Genome-based phylogeny and tree construction was carried out by the VICTOR web service (66) between the CI00795 prophage and all 27 members of the *Casadabanvirus* genus, as currently listed with the International Committee on Taxonomy of Viruses (https://ictv.global). Reference genomes for 27 *Casadabanvirus* phages were download from the NCBI and reannotated along with the CI00795 prophage using pharokka (61) to standardize annotations across all genomes. Whole-genome pairwise comparisons of amino acid sequences were conducted using the Genome-BLAST Distance Phylogeny method (67) using the d6 formula. Resulting intergenic distances were used to infer a balanced minimum evolution tree with branch support by FASTME 2.0, including SPR postprocessing (68), with 100 pseudo-bootstrap replicates each. Trees were rooted at the midpoint and visualized with ggtree (69). Taxon boundaries at the species, genus, and family levels were estimated with the OPTSIL program (70) with a recommended clustering thresholds and F value of 0.5. Homology searches were conducted with HHPRED (71) and PHYRE2 (72), and functional domain predictions were performed using SMART (73).

### Intraspecies antagonism assays

In order to establish a baseline of inhibitory activity between *Pseudomonas* strains used in Appelmans and screen for resident prophage, intraspecies antagonism assays were conducted. Each bacterial host used in Appelmans was cross-tested for intraspecies inhibitory activity (PAO1, PA103 PA14, PAK, PD0300, CI00780, CI00795, and CI01788). Bacterial cultures were grown and exposed to ultraviolet light or mitomycin C (final concentration 1.0 μg/mL) and incubated overnight; an uninduced culture was included as a control. The cultures were then centrifuged at 5,000 rpm for 15 minutes to pellet cellular debris, and supernatants were filter-sterilized (0.2 μm). Filter-sterilized samples were then tenfold serially diluted and spotted at a volume of 10 μL onto lawns of all bacterial hosts included in Appelmans. Plates were then incubated at 37°C overnight and observed for inhibitory activity and plaque formation.

### CI00795 prophage host-range determination by PCR

In order to establish the initial host range of the wild-type *Casadabanvirus* prophage and distinguish observed plaque formation on the five laboratory strains between the *Casadabanvirus* prophage and the *Hollowayvirus* prophage of strain CI00795, unique PCR primers were designed to target the large terminase subunit of each prophage, which has classically been used to distinguish phages across clades (74–77). The forward and reverse primer sequences used to target the *Casadabanvirus* prophage in this study were 5’ CTAGCGTTGGTTAGAAGCCA 3’ and 5’ CGCCAGTGTCAAAAGAATCG 3’, respectively. The forward and reverse primer sequences for the *Hollowayvirus* prophage were 5’ CGAGTGACCACCTTCGTC 3’ and 5’ CTCACAGTCGCCAGTCAG 3’, respectively (primers were ordered from Invitrogen). To determine the host range of each prophage, 100 μL of the supernatant from strain CI00795 was added to 3 mL of molten LB top agar, along with 100 μL of the overnight culture for each of the five *P. aeruginosa* laboratory hosts. Plates were allowed to incubate overnight at 37°C and observed for plaque formation. Individual plaques were then harvested from each bacterial lawn and screened by PCR, as previously described (78). Briefly, plaques were harvested by picking an isolated plaque using a 1,000-μL pipette tip and resuspending the plaque in 50 uμL of ultrapure water. Samples were vortexed and allowed to sit at room temperature for 30 minutes. Samples were then placed in a heat block at 98°C for 5 minutes, then vortexed, and centrifuged at 5,000 × *g* for 5 minutes. Samples were then used as the DNA template for PCRs using standard *Taq* DNA polymerase (NEB) according to the manufacturer’s instructions. Each plaque sample was screened using both PCR primer sets to ensure no double positives occurred.

## Data Availability

Raw read sequencing data for clinical isolate CI00795, input phages D3, M6, and JM2, and ten output phages are available under NCBI BioProject accession number PRJNA1104273. The assemblies for CI00795 and input phages D3, M6, and JM2 are available under the GenBank accessions CP158022, PP944329, PP944330, and PP944331, respectively.

## SUPPLEMENTAL MATERIAL

Supplemental material aem.00884-24-s0001.pdf Figures S1 to S4; Tables S1 to S3.

ASM does not own the copyrights to Supplemental Material that may be linked to, or accessed through, an article. The authors have granted ASM a non-exclusive, world-wide license to publish the Supplemental Material files. Please contact the corresponding author directly for reuse.
