# Supplementary material for: Directed evolution of bacteriophages: thwarted by prolific prophage
Source: Appl Environ Microbiol. 2024 Oct 30;90(11):e00884-24. doi: 10.1128/aem.00884-24 (PMC11577772; doi:10.1128/aem.00884-24)
Supplement: Supplemental material — Figures S1 to S4; Tables S1 to S3. [file aem.00884-24-s0001.pdf]

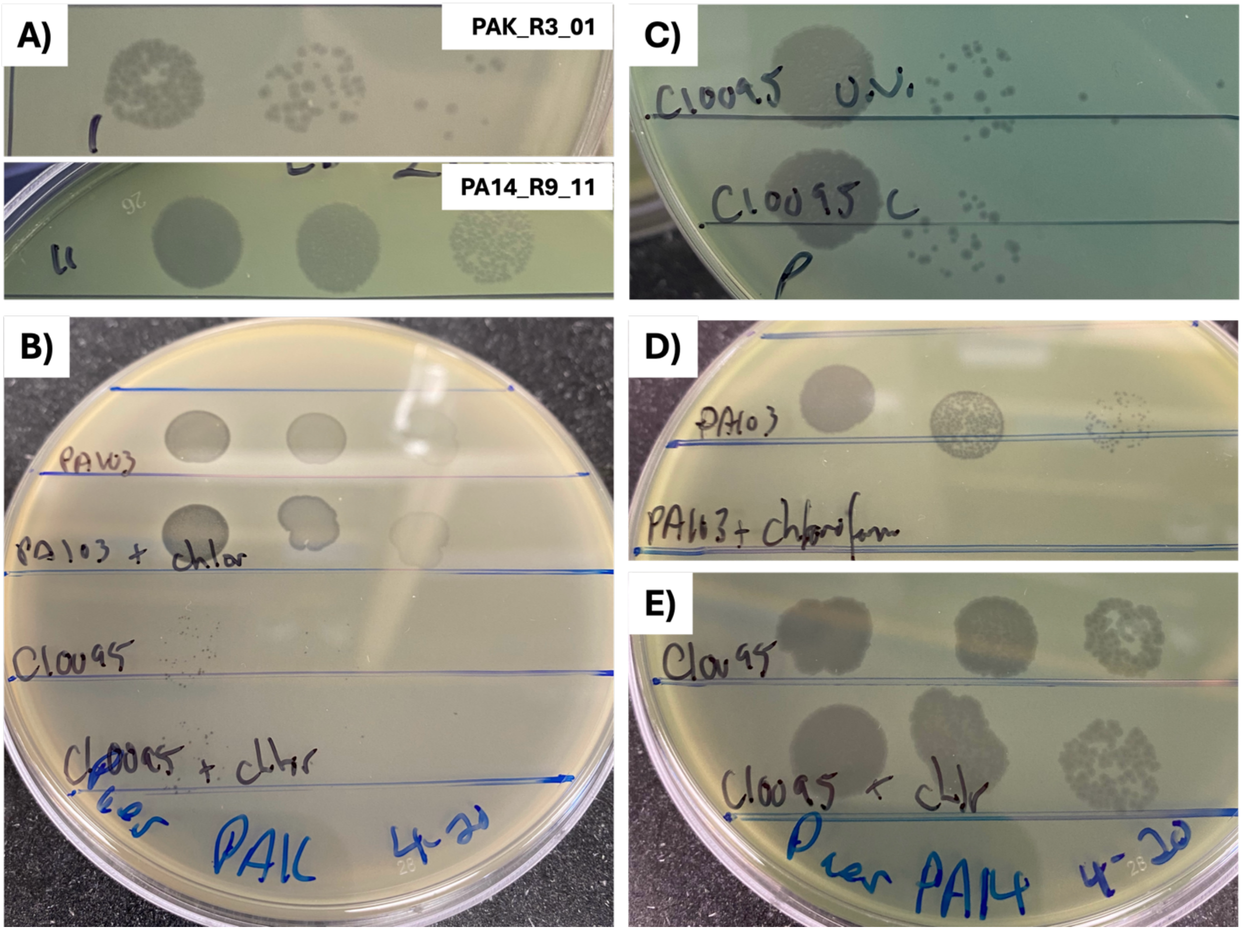

Supplemental Figure 1. Examples of plaque formation and inhibitory activity from lysate that has been ten-fold serially diluted and spotted in 10ul volumes onto lawns of *P. aeruginosa*. **A)** Examples of plaque formation on non-permissive host PA14 from two output phages PAK_R3_01 and PA14_R9_11. **B)** Examples of inhibitory activity (from PA103 supernatant) and plaque formation (from CI00795 supernatant) on strain PAK**; note** how PA103 supernatant does not resolve into individual plaques at higher dilutions, which is characteristic of tailocin (aka pyocin) activity. **C)** Exposure of cultures to ultraviolet light (CI00795 u.v.) did not increase level of plaque formation compared to control (CI00795 c). **D)** Example of plaque formation from PA103 supernatant on strain PAO1; exposure to chloroform eradicated plaque formation. **E)** Example of plaque formation from CI00795 supernatant on host strain PA14; exposure to chloroform had no effect.

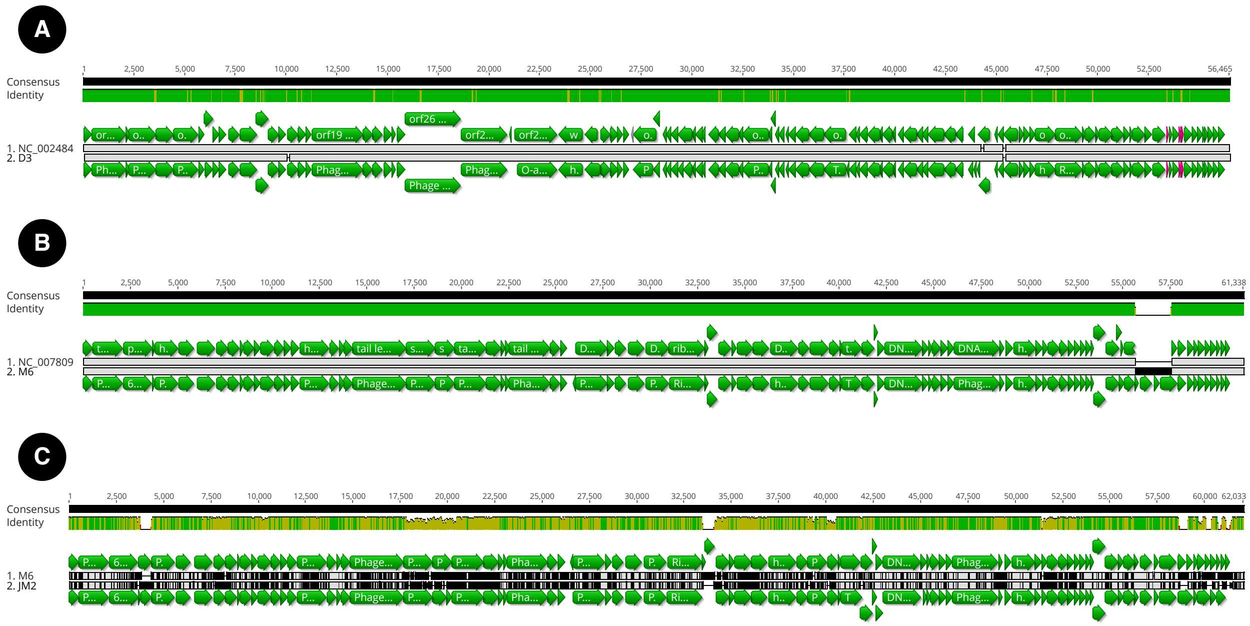

Supplemental Figure 2. Genome maps and pairwise alignments of input parental phages **A**) D3 with D3 reference genome NC_002484, **B**) M6 with M6 reference genome NC_007809, and **C**) JM2 aligned with M6.

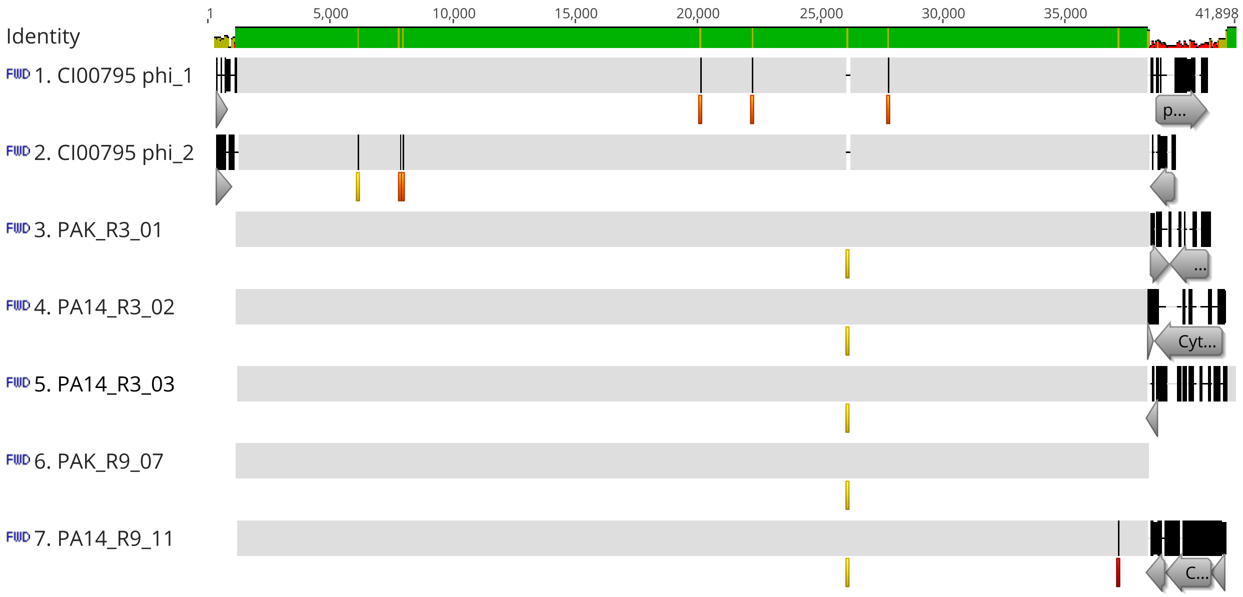

Supplemental Figure 3. Multiple sequence alignment of two integrated *Casadabanvirus* prophages (phi_1 and phi_2) from CI00795 and five prophage-derived output phages isolated from the Appelmans experiment. Gray arrows indicate heterogenous bacterial host DNA, yellow tick marks indicate synonymous and intergenic mutations, orange tick marks indicate nonsynonymous mutations between the two integrated prophages (phi_1 and phi_2), red tick mark indicates unique nonsynonymous mutation (PA14_R9_11).

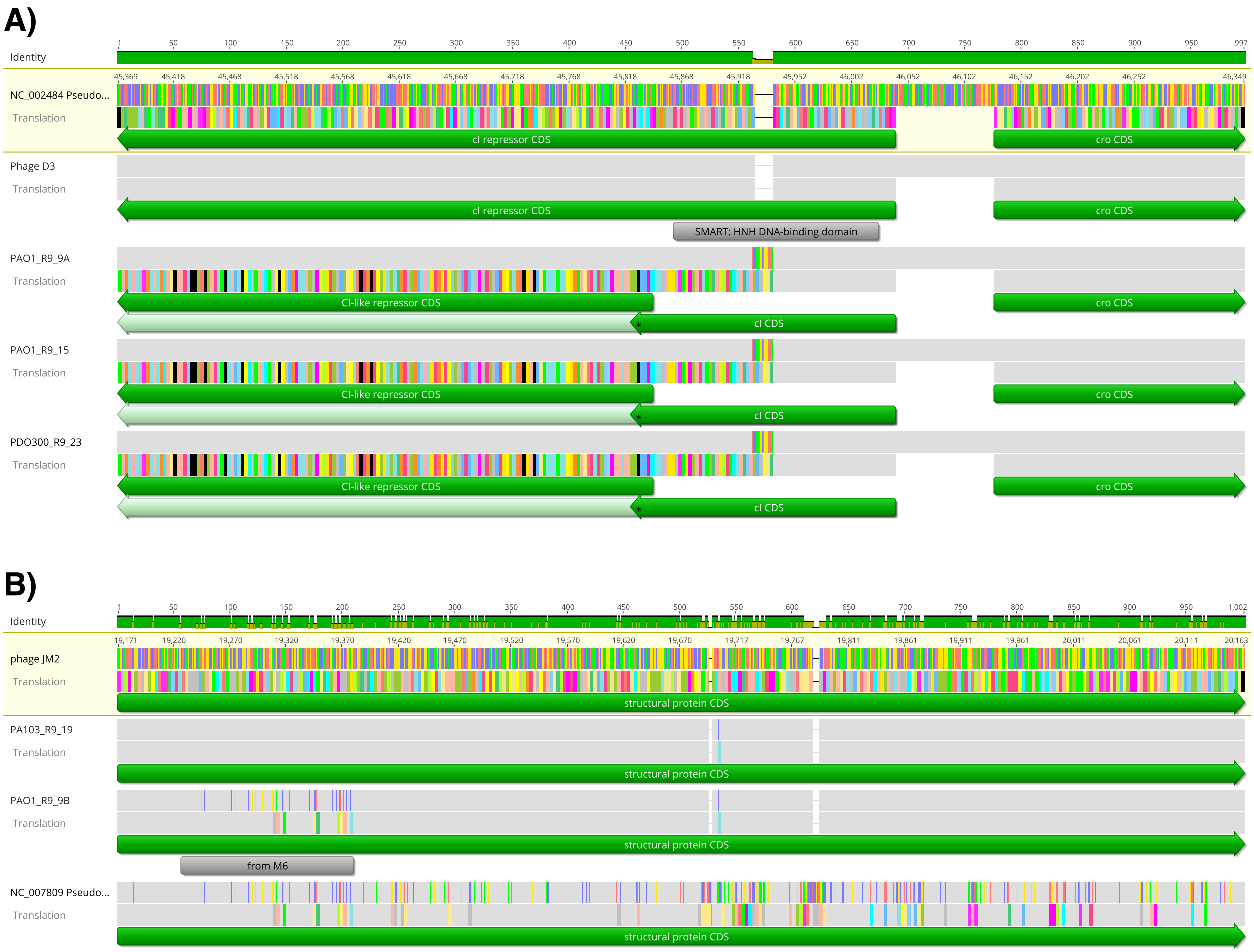

Supplemental Figure 4. Multiple sequence alignments of Appelmans output phages derived from input phages. **A)** D3 derived output phages aligned with input phage D3 and the D3 RefSeq genome NC_002484, showing the cI/cro repressor genes (D3p074). Output phages PAO1_R9_9A, PAO1_R9_15, and PDO300_R9_23 all shared a 19bp insertion in the N-terminal of the cI repressor gene, altering the open reading frame. SMART predicted DNA-binding functional domain is annotated (residues 6-60). **B)** JM2 derived output phages aligned with input phage JM2 and M6 RefSeq genome NC_007809, showing the 154 bp region of recombination in the N-terminal of the structural virion protein (locus tag: PPM6_gp033) between JM2 and M6 in output phage PAO1_R9_9B, resulting in nine amino acid changes in that region. One amino acid substitution is also depicted for both PAO1_R9_9B and PA103_R9_19 at residue 178/331 (Ser->Arg).

Supplemental Table 1. Variant analysis of the *Casadabanvirus* prophages integrated at two loci in the genome of bacterial host *P. aeruginosa* strain CI00795 (referred to as phi_1 and phi_2) and output prophage derived samples isolated from the Appelmans experiment.

**Nucleotide position***	**gene**	**nucleotide**		**residue**	**amino acid**	
**Prophages integrated into CI00795 genome**						
		phi_1	phi_2		phi_1	phi_2
5,002	DNA transposition protein	G	A	110	synonymous	
6,681	DNA binding protein	A	G	175	Ile	Val
6,822	transmembrane/signal peptide domain contain protein	A	G	15	Asp	Gly
18,945	type I-E anti-CRISPR protein	A	G	44	Ser	Gly
21,057	Mu-like-gpT major capsid protein	A	G	72	Ser	Gly
26,638	Tape measure protein	A	G	556	Thr	Ala
**Unique mutations in output phages derived from prophages**						
		**Ref**	**Five output phages**			
24,939	Intergenic <- tape measure protein	GGGGG	G+GGGGG	N/A	N/A	N/A
		**Ref**	**PA14_R9_11**			
35,980	Putative tail fiber protein	G	A	203	Asp	Asn

*in reference to genome of CI00795 phi_1 (integrated at nucleotide position 771,168 -> 808,37 of Genbank Accesion CP158022)

Supplemental Table 2. Results from PCR verification of the CI00795 prophages host range based individual testing of plaques with both primer sets, with no evidence or double positives.

Prophage Target*	*Pseudomonas aeruginosa* host strains				
	PAO1	PAK	PA103	PA14	PDO300
Casadabanvirus	+	+	+	+	+
Hollowayvirus	--	+	--	--	+

*Primers were unique to the large terminase subunit of either prophage.

Supplemental Table 3. Bacterial and bacteriophage strains used in this study.

Bacterial Strain	Source
PAO1 Seattle*	Pradeep Singh, University of Iowa Eckstein Medical Research Building
PAO1 Fralick	Joe A. Fralick, Department of Immunology and Molecular Microbiology, Texas Tech University Health Sciences Center
PA103	Pradeep Singh, University of Iowa Eckstein Medical Research Building
PA14	Joe A. Fralick, Department of Immunology and Molecular Microbiology, Texas Tech University Health Sciences Center
PAK	Pradeep Singh, University of Iowa Eckstein Medical Research Building
PDO300	Pradeep Singh, University of Iowa Eckstein Medical Research Building
CI00780	Texas Department of Health, Jim Bull
CI00795	Texas Department of Health, Jim Bull
CI01788	Texas Department of Health, Jim Bull,
**Phage Strain**	
D3**	ATCC BAA-47-B1
M6	Received from Denise Tremblay at the University of Laval Oral Ecology Research Group
JM2	Isolated from Moscow, ID sewer sample May 2005

*Used as phage propagation host strain for all input phage, not used in Appelmans
**This strain was listed as F116 when ordered from ATCC; sequencing confirmed it as phage D3.
